# Primordial odontogenic tumor: A systematic review

**DOI:** 10.4317/medoral.23432

**Published:** 2020-02-10

**Authors:** Ronell Bologna-Molina, Vanesa Pereira-Prado, Celeste Sánchez-Romero, Rogelio González-González, Adalberto Mosqueda-Taylor

**Affiliations:** 1 Molecular Pathology Area, School of Dentistry, Universidad de la República, Montevideo, Uruguay; 2 Department of Research, School of Dentistry, Universidad Juárez del Estado de Durango, Durango, México; 3 Health Care Department, Universidad Autónoma Metropolitana, Ciudad de México, México

## Abstract

**Background:**

The primordial odontogenic tumor (POT) is a recently described benign entity with histopathological and immunohistochemical features suggesting its origin during early odontogenesis. Aim: To integrate the available data published on POT into a comprehensive analysis to better define its clinicopathological and molecular features.

**Material and Methods:**

An electronic systematic review was performed up to September 2019 in multiple databases.

**Results:**

A total of 13 publications were included, representing 16 reported cases and 3 molecular studies. The mean age of the affected patients was 11.6 years (range 2-19), with a slight predominance in males (56.25%). The posterior mandible was the main location (87.5%), with only two cases affecting the posterior maxilla. All cases appeared as a radiolucent lesion in close relationship to an unerupted tooth. Recurrences have not been reported to date. Microscopically, POT comprises fibromyxoid tissue with variable cellularity surrounded by a cuboidal to columnar odontogenic epithelium but without unequivocal dental hard tissue formation. A delicate fibrous capsule surrounds (at least partially) the tumor. The epithelial component shows immunohistochemical positivity for amelogenin, CK19, and CK14, and variable expression of Glut-1, Galectin-3 and Caveolin-1, Vimentin, p-53, PITX2, Bcl-2, Bax and Survivin; the mesenchymal tissue is positive for Vimentin, CD90, p-53, PITX2, Bcl-2, Bax, and Survivin, and the subepithelial region exhibits the strong expression of Syndecan-1 and CD34. The Ki-67 index is low (<5%). The negative or weak expression of dentinogenesis-associated genes could explain the inhibition of dentin and subsequent enamel formation in this neoplasm.

**Conclusions:**

POT is an entity with a well-defined clinicopathological, immunohistochemical and molecular profile that must be properly diagnosed and differentiated from other odontogenic lesions and treated consequently.

** Key words:**Primordial odontogenic tumor, systematic review.

## Introduction

**Figure 1:**
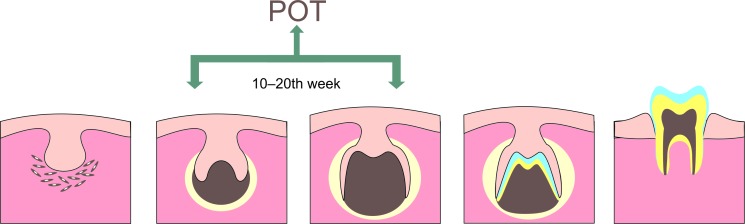
Process of odontogenesis and suggested location of primordial odontogenic tumor (POT) origin according to developmental stages: late cap and early bell.

In 2014, the primordial odontogenic tumor (POT) was described for the first time (1), and subsequently this entity was included in the World Health Organization (WHO) Classification of Head and Neck Tumours in the group of benign mixed neoplasms (2). The name was coined due to its possible development from the early stages of odontogenesis (Fig. 1).

Since its original description in 2014, sixteen cases of POT have been reported in the literature, presenting as a well-defined radiolucent lesion in close proximity to the crown of an unerupted tooth, producing bone expansion, radicular resorption and tooth displacement of variable extent (3-9).

The aim of this systematic review is to collect and integrate the available data published on POT into a comprehensive analysis to better define its clinicopathological, radiological and molecular features.

## Material and Methods

This study followed the PRISMA (Preferred Reporting Items for Systematic Reviews and Meta-analyses) guidelines (10).

- Search strategies

An electronic search was performed up to September 2019, with date restriction since 2014 (first description of the POT). The following databases were accessed: PubMed/MEDLINE, Cochrane, and SpringerLink. The search strategy used in all databases consisted of the following keywords: primordial odontogenic tumor.

- Inclusion and exclusion criteria

The inclusion criteria were as follows: 1) cases diagnosed as POT with sufficient clinical, microscopic, and immunohistochemical information to confirm a definite diagnosis based on the WHO histological classification of odontogenic tumors (2), including case reports and molecular studies, 2) articles in English language, and 3) articles included in PubMed/MEDLINE, Cochrane, and SpringerLink databases.

The exclusion criteria were as follows: 1) case reports without sufficient information to confirm a definite diagnosis, 2) book chapters, reviews and meta-analyses, 3) documents in a different language from English, or 4) articles published before 2014.

- Study selection

**Figure 2:**
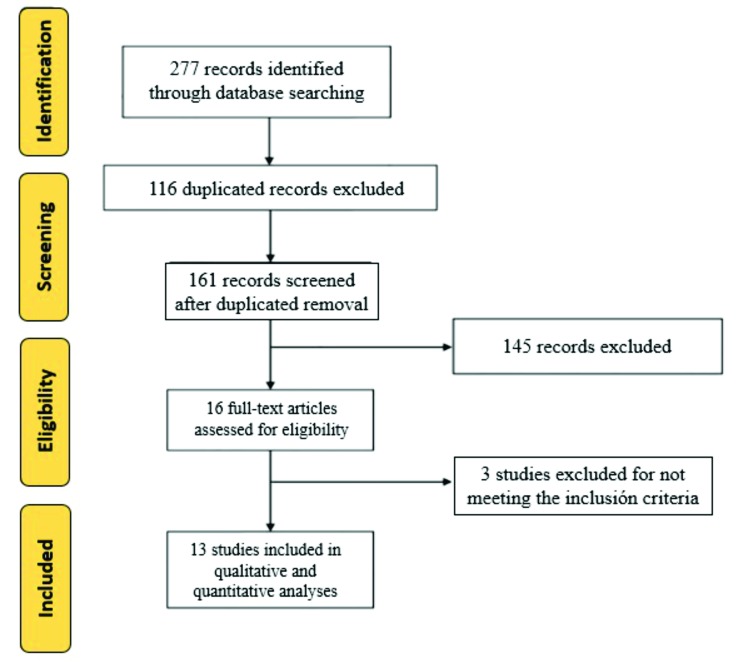
Study selection flowchart, according to PRISMA guidelines.

The titles and abstracts of all studies found in the database search were independently reviewed by two authors; subsequently, the articles that met the inclusion criteria and those lacking information in the title or abstract were completely evaluated using the EBLIP Critical Appraisal Checklist. The studies selected by each author were crossed to ensure that they were properly chosen, according to the inclusion criteria and the checklist (Fig. 2).

- Data extraction

All relevant information, such as reference, year of publication, patient nationality or country of publication, number of cases, patient sex and age, tumor location, signs and/or symptoms, imaging presentation, treatment, follow-up, immunohistochemical and molecular features, were extracted (when available) using a specific Table.

- Analysis

The abovementioned data were analyzed with descriptive statistics. Since POT is a benign tumor with indolent behavior and no recurrences reported to date, it was not necessary to perform statistical tests to evaluate the association of clinicopathological variables with prognosis or survival.

## Results

- Literature search

The process of searching and screening the articles is summarized in Fig. 2. The initial search recorded a total of 277 articles; of these, 116 articles were excluded due to duplication. Subsequently, two reviewers, independently, with a substantial concordance indicated by a kappa coefficient of k = 0.76, assessed the titles and abstracts, resulting in the exclusion of 146 publications. In the evaluation of the 16 remaining papers, 2 were excluded because they were reviews and 1 was excluded for not meeting the criteria to confirm the diagnosis of POT.

- Description of the selected studies

A total of 13 publications were included, representing 16 reported cases and 3 molecular studies (Table 1). The studies and cases originated from nine countries. The study of Mosqueda-Taylor *et al*., 2014 included six cases from four different countries (México, Guatemala, Brazil and Spain), and additional cases were reported in the U.S.A., Japan, Egypt, India, México, Brazil and the Philippines (Table 2).

- Descriptive analysis of the clinical and radiographic features

The age of the patients varies in a range of 2-19 years old, with a mean of 11.6. Males were slightly more affected (56.25%) with a male to female ratio of 9:7. All cases occurred in the posterior region of the jaw, mainly in the mandible (87.5%). All patients were asymptomatic, with most presenting swelling in the affected region, and in only two cases, the tumor was discovered as an incidental radiographic finding (4,8). Radiographically, all cases were associated with at least one unerupted tooth, with the majority presenting as unilocular and well-defined radiolucencies, and approximately one-third of the cases exhibiting biloculated or multiloculated appearances. Tooth displacement and root resorption were commonly found (in 75% and 87.5% of cases, respectively). Most of these cases involved unerupted teeth, particularly the third molar (62.5%), followed by the second deciduous molar. The size of the lesions ranged from 9 to 90 mm; however, most of these anomalies were ≥ 30 mm, with a mean of 41 mm (Table 2, Table 3).

**Table 1:**
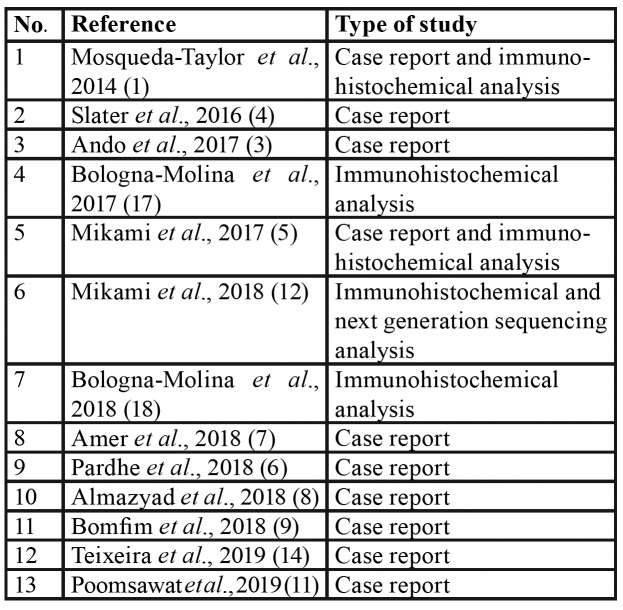
Selected articles for the systematic review.

**Table 2:**
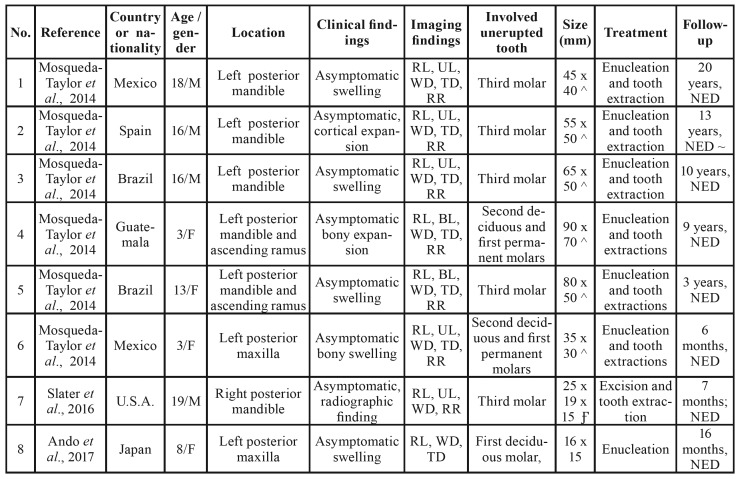
Clinicopathological and radiographic features of primordial odontogenic tumor.

**Table 2 cont.**
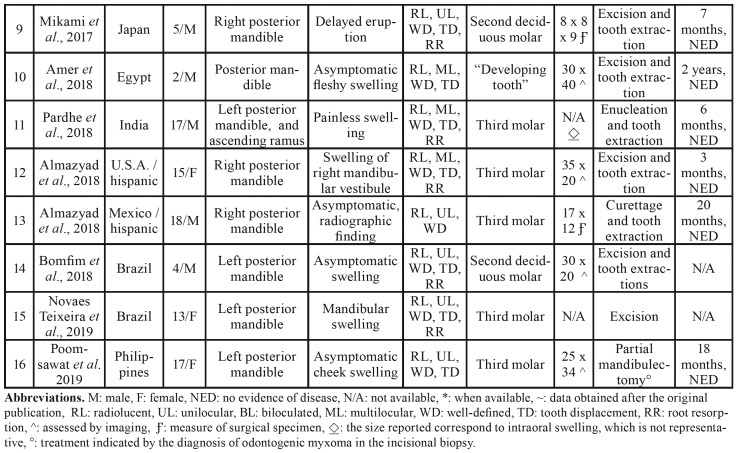
Clinicopathological and radiographic features of primordial odontogenic tumor.

**Table 3:**
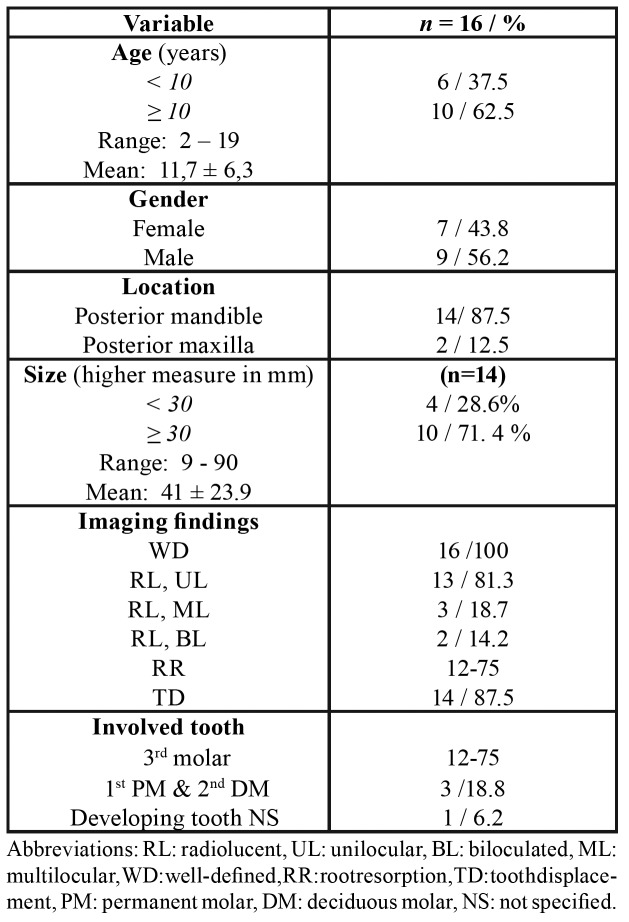
Summary of clinical data of POT.

All cases, except one, were treated with simple excision/enucleation with extraction of the involved teeth and to date, there are no reports of recurrences (follow-up range: 3 months to 20 years) (Table 2). One case was treated with a partial mandibulectomy due to a misdiagnosis of odontogenic myxoma in the incisional biopsy (11). Macroscopically, POT is a solid, multilobulated whitish and glossy mass, without cystic spaces, and this tumor is surrounded by a capsule or at least well demarcated from the surrounding structures (1,2,9). In some cases, the dental follicle can be identified in the surgical specimen with a dark reddish color (4,5,9).

The demographic, clinicopathological and radiographic features of the 16 POT cases are summarized in Table 2, and the descriptive statistics are summarized in Table 3.

- Histopathological features

**Figure 3:**
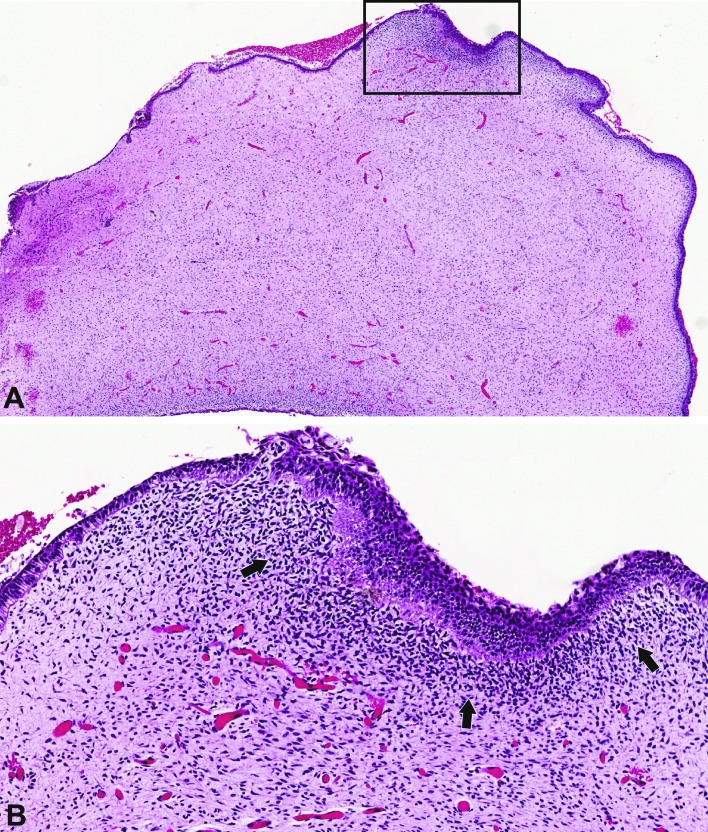
Histological features of POT. A: The fibromyxoid mesenchymal tissue is completely surrounded by cuboidal to columnar odontogenic epithelium. A subepithelial cellular condensation of the mesenchyme is observed; additionally, higher vascularization is observed adjacent to this region. B: In this magnified region, the tumor is covered by an odontogenic epithelium with ameloblastic and cuboidal morphology. The subepithelial mesenchymal condensation (arrows) is observed next to a prominent vascularized area (hematoxylin and eosin staining, zoom of digitalized slide A: 2x, B: 20x).

POT comprises mesenchymal fibromyxoid tissue with variable cellularity that in most cases resembles the dental papilla, surrounded at the periphery by a cuboidal to columnar epithelium similar to the inner epithelium of the enamel organ. Occasionally, suprabasal stellate reticulum-like areas may be observed. The tumor is at least partially enclosed by a thin fibrous capsule (Fig. 3). A condensation of mesenchymal cells in the subepithelial region is also commonly observed (Fig. 3). Invaginations of the surrounding epithelium can be focally present within the mesenchymal component, resulting in ameloblastic fibroma-like islands on tangential sections.

This tumor is devoid of dental hard tissues; nevertheless, four authors have described the presence of small foci of calcifications within the epithelial tissue, specifically, the stellate reticulum-like areas. However, no evidence of odontoblastic differentiation or the induction of dental hard tissue deposition has been described thus far. These intraepithelial calcifications are round small masses of hard material with a globular or concentric appearance (5,8,9,11).

- Immunohistochemical features

The epithelial component shows consistent immunohistochemical positivity for CK14, CK19, amelogenin, ameloblastin and dentin sialophosphoprotein precursor (DSPP). However, other markers, such as Glut-1, Vimentin, MOC-31, Caveolin-1, Galectin-3, PITX2, p53, Bax, Bcl-2, Survivin and *PTEN*, are variably and focally expressed. The mesenchymal tissue is strongly positive for Vimentin, Syndecan-1, PITX2, Endoglin (CD105), CD34, Cyclin D1, Bax, Bcl-2, Survivin and p53, while *PTEN*, CD90 and DSPP were detected with moderate positivity. The proliferation index assessed by Ki-67 is <5%. The condensed mesenchymal tissue adjacent to the epithelium presents the strong expression of CD34 and Syndecan-1. This tumor is negative for BRAF V600E and calretinin by immunohistochemistry.

- Molecular characterization

To date, 151 cancer- and 42 odontogenesis-associated genes have been analyzed in POT by next-generation sequencing, and no mutations were detected. Nevertheless, the expression of the dentinogenesis-associated genes Bglap, Ibsp and Nfic was negative or very weak. DSPP mRNA is highly expressed in POT (12).

## Discussion

Although POT is a recently described and accepted entity (1,2), this systematic review showed that the 16 cases reported to date exhibit a well-defined profile of clinicopathologic, radiographic, immunohistochemical and molecular features that can be summarized as follows: this tumor occurs in the first and second decades of life, affecting the posterior region of the jaw, particularly the mandible. Radiographically, POT appears as a well-defined radiolucent unilocular lesion always associated with unerupted teeth, such as deciduous or third molars, with a mean size of 4.1 cm, showing tooth displacement and frequent root resorption (Table 1, Table 2).

Considering that most lesions appear as unilocular radiolucencies in close relationship with the crown of an unerupted tooth, the main radiographic differential diagnosis includes a dentigerous cyst and an ameloblastic fibroma; however, multilocular lesions with tooth displacement and root resorption could mimic other odontogenic tumors and cysts, such as ameloblastoma, odontogenic myxoma and odontogenic keratocyst (2,4,11). POT is a benign tumor with an indolent course, as there are no reported recurrences after conservative excision (Table 1).

Macroscopically, POT is a solid, multilobulated whitish and glossy mass with no evidence of cystic changes, and this tumor is well demarcated from the surrounding structures. Considering these aspects, it is convenient to rule out the possibility of ameloblastic fibroma in excision specimens and of odontogenic myxofibroma in incisional biopsies (1,2,9,11).

Microscopically, POT essentially shows mesenchymal fibromyxoid tissue resembling in large areas the dental papilla, surrounded by a cuboidal to columnar epithelium that resembles the inner epithelium of the enamel organ (Fig. 3) (2,4,5). These features differ from their main histopathological differential diagnoses: ameloblastic fibroma, hyperplastic dental follicle and odontogenic myxoma (1,4). In the last case, it is mandatory to identify the highly distinctive features of POT to avoid the misdiagnosis of odontogenic myxoma, particularly in small incisional biopsies and subsequent overtreatment, a situation that has been reported previously (11).

In a representative biopsy or a complete surgical specimen of POT, it is possible to exclude an odontogenic myxoma, since this tumor is not surrounded by an odontogenic epithelium, and to differentiate it from an ameloblastic fibroma, which presents cords and islands of odontogenic epithelium within the dental papilla-like myxoid stroma (2). Some authors have stated that the areas of ameloblastic epithelium of POT are similar to those of the lining of unicystic ameloblastoma (4); however, POT presents as a tumor mass and not as a cystic lesion. Additionally, the differences in the mesenchymal component (fibrous tissue for ameloblastoma and fibromyxoid for POT) are helpful in confirming the diagnosis (Fig. 3) (2,4).

We excluded a recently published POT case report from this study because most of the clinicopathological and radiographic characteristics did not match the profile of the POT, such as its location (anterior), lack of association with an unerupted tooth, small size, subepithelial odontoblastic differentiation, and dentinoid production (13). Consequently, we consider that this case represents a developing odontoma, which must be included as another differential diagnosis of POT, mainly in very small lesions in children. In contrast to odontoma, POT does not present the production of dental hard tissues or odontoblastic differentiation (Table 2) (3,5,7,14).

Initially, the term POT was coined due to the histologic resemblance of the tumor with the appearance of the epithelial and mesenchymal elements during the early stages of tooth development (Fig. 3), when the epithelium is located peripherally, mimicking the relation between the inner epithelium of the immature enamel organ and the primitive dental papilla, without inductive effects over the ectomesenchyme (Fig. 2) but lacking the ability to follow a normal evolution toward histo- and morphodifferentiation (1,12). Heterogeneity in epithelial differentiation within the same tumor has been documented previously by histopathological analysis, describing features, such as localized areas of ameloblastic differentiation(3,4) and focal stratum intermedium-like structures, as well as some superficial stellate reticulum-like areas, which are not necessarily representative of the findings of POT (5,9). The histomorphological variability may reflect the heterogeneity of the tumor caused by, for example, slight differences in the state of differentiation among the reported cases (5,12).

Although POT is devoid of dental hard tissues, four cases showed small foci of calcifications within the epithelial layer (5,8,9,11), which may suggest that alkaline phosphatase could play a role in the formation of these calcifications, as occurs in the calcifying epithelial odontogenic tumor (15,16).

Immunohistochemically, POT has shown a low proliferation index (<5%), which defines this lesion as a slow-growing benign tumor in which cell proliferation does not seem to be the main mechanism implicated in tumoral growth and expansion (17). Nonetheless, one study has shown that the condensed subepithelial region exhibits higher vascularization and positivity for Glut-1 and antiapoptotic markers, suggesting a major participation of metabolic, antiapoptotic and angiogenic events in tumor growth (5,17).

The immunoexpression patterns described in POT corresponded to those found in the normal early stages of tooth development: the condensed mesenchymal subepithelial area seems to be the most proliferative and shows high expression of Syndecan-1 and CD34, which, along with the epithelial expression patterns of CK14, CK18 and CK19, and focal PITX2, as well as the mesenchymal and epithelial staining for vimentin, resembles the initial period of tooth formation, suggesting that POT origins around the 10th to 20th week of embryonal development (cap stage to late bell stage of tooth germ) (Fig. 2) (1,5,12,18).

In fact, the epithelial portion of the tumor consistently expresses CK14 and CK19, while other markers, such as Vimentin, Amelogenin, Glut-1, MOC-31, and Caveolin-1. Galectin-3, PITX2, p53, Bax, Bcl-2, Survivin and *PTEN*, are variably expressed in focal areas, suggesting a dynamic tissue presenting cells in different stages of maturation and exhibiting a transition between early stages of tooth development to those with ameloblastic maturation but without induction of odontoblastic maturation or production of mineralized tissues (5,17,18). This immunohistochemical profile supports the denomination of “primordial” for this tumor.

Recently, somatic mutations of cancer-associated genes, such as BRAF V600E (19), SMO (20), KRAS (21), PTCH1 (22) and CTNNB1 (23,24), have been reported in some benign odontogenic tumors and cysts. These findings indicate that neoplastic proliferation of some odontogenic tumors may be triggered by genetic alterations affecting oncogenic signaling pathways (25). As odontogenic tumors are derived from the cells of the tooth-forming apparatus and their remnants (2), the evaluation of odontogenesis-associated genes is also important to elucidate the pathogenesis of POT. The absence of immunoexpression of the mutant protein BRAF V600E confirms that this mutation is not implicated in the pathogenesis of POT, excluding it from the category of the BRAF mutated ameloblastic tumors (17).

In the study of Mikami *et al*., [2018], no somatic mutations were detected when 151 cancer- and 42 odontogenesis-associated genes were examined, and the mRNA expression level of odontogenesis-associated genes in POT was determined by next-generation sequencing. Nevertheless, the expression of the dentinogenesis-associated genes Bglap, Ibsp and Nfic was negative or very weak, likely due to epigenetic silencing mechanisms, explaining the inhibition of dentin (and consequently, enamel) formation in POT (12).

In tooth germ development, DSPP is immunohistochemically positive in preameloblasts and preodontoblasts (26). Although DSPP is immunoexpressed in the epithelium and mesenchyme of POT, and DSPP mRNA is also highly expressed, neither odontoblast differentiation nor the induction of dentin formation is morphologically observed (12,18).

In brief, with the data obtained by immunohistochemical and genetic studies, we can conclude the following: POT is a benign, slow-growing odontogenic neoplasm, which shows a low proliferation rate and moderate vascularization (17). The epithelial tissue surrounding the tumor mass is nonstatic and shows varying degrees of maturation, with a transition from an inner enamel epithelium morphology to areas of ameloblastic maturation, but without evidence of induction leading to mineralized tissue production (5,17). The subepithelial area shows the expression of several proteins, suggesting that it is a highly active tumor region (17,18). The pathogenesis of the tumor does not appear to be linked with any type of known gene mutation; however, there is an inhibition of enamel and dentin formation by the downregulated expression of genes and proteins associated with dentinogenesis (12).

In summary, in this systematic review, we showed that the 16 cases reported to date exhibit a well-defined profile of clinicopathologic, radiographic, immunohistochemical and molecular features. Despite the indolent clinical course of POT, it is crucial to identify its highly distinctive presentation to avoid misdiagnosis, mainly in small incisional biopsies and subsequent overtreatment.
